# Unveiling Species Diversity Within Early-Diverging Fungi from China XII: Six New Species of *Mucor* (*Mucoromycota*)

**DOI:** 10.3390/jof12020098

**Published:** 2026-01-30

**Authors:** Wen-Xiu Liu, Fei Li, Zi-Ying Ding, Xin-Yu Ji, Shu-Ting Geng, Hong-Yu Zou, Heng Zhao, Shi Wang, Xiao-Yong Liu

**Affiliations:** 1 College of Life Sciences, Shandong Normal University, Jinan 250358, China; 18054356852@163.com (W.-X.L.);; 2 CAS Key Laboratory of Forest Ecology and Silviculture, Institute of Applied Ecology, Chinese Academy of Sciences, Shenyang 110016, China; zhaoheng181@mails.ucas.ac.cn; 3 Institute of Microbiology, Chinese Academy of Sciences, Beijing 100101, China

**Keywords:** Mucorales, morphological taxonomy, molecular phylogeny, new taxa, fungal diversity

## Abstract

*Mucor* is widely distributed in nature and extensively applied in industries, food, and other fields. Based on analyses of the internal transcribed spacer (ITS) and the large subunit (LSU) of the ribosomal RNA gene, RNA polymerase II largest subunit gene (*RPB1*), and their morphological characteristics, six new species of *Mucor* were identified. *Mucor catenatus* sp. nov. forms chlamydospores in chains. *Mucor jujubinus* sp. nov. is characterized by jujube-shaped columellae. *Mucor macrosporangium* sp. nov. produces larger sporangia. *Mucor multiramosus* sp. nov. features extensively branched sporangiophores. *Mucor oligorhizus* sp. nov. shows a rare rhizoid formation. *Mucor tumidus* sp. nov. usually develops swellings in sporangiophores. This study represents the twelfth contribution in a series exploring early-diverging fungal diversity in China and raises the number of accepted *Mucor* species to 60.

## 1. Introduction

*Mucor* belongs to Mucoraceae, Mucorales, Mucoromycetes, and Mucoromycota [1]. It is widely distributed in environments such as soil, feces, grasses, air, rotten fruits, vegetables, and cheese [2,3,4,5,6,7,8]. It is an important decomposer in nature, capable of secreting a variety of enzymes to break down organic substances like carbohydrates, proteins, and fats, thereby participating in the material cycle in nature [9,10]. Many species of this genus play significant industrial roles by producing amylases, proteases, lipases, and organic acids such as lactic acid and fumaric acid [11,12]. Certain *Mucor* species are also widely used in traditional Asian food fermentation due to their abundant production of proteases and lipases [13,14,15,16,17,18,19]. However, members of this genus are also prone to contaminating carbohydrate-rich foods, resulting in food mold and spoilage [20]. Some members are opportunistic mucormycosis pathogens [21,22,23,24], especially when the body’s immune system is compromised and the individual is extremely debilitated, leading to lesions in sites such as the nose, brain, lungs, and gastrointestinal tract [25,26].

The genus was first described by Fresenius in 1850 [27]. As an early-diverging fungal lineage, it is characterized by coenocytic hyphae that develop septa only during reproduction or in aging mycelia, along with polymorphic sporangiophores and terminal spherical sporangia. In a recent study by Nguyen et al., the number of accepted species in this genus was determined to be 54 [28].

During the isolation of fungi from leaf and soil samples collected in Hainan, Xizang, Guangxi, and Yunnan in China, fifty-nine *Mucor* strains were found to differ from other known species in terms of their molecular and morphological characteristics. These strains are classified into six new species and described herein. This is the twelfth report of a serial work on diversity of Chinese early-diverging fungi [29,30,31].

## 2. Materials and Methods

### 2.1. Sample Collection and Strain Isolation

During 2024–2025, soil samples were collected from four regions in China, namely Hainan Province, Xizang Autonomous Region, Guangxi Zhuang Autonomous Region, and Yunnan Province. Strain isolation was conducted following the plate dilution method and single spore isolation technique as described in previous studies [32,33], with the specific procedures outlined below. First, 1 g of each soil sample was accurately weighed and homogenized in 10 mL of sterile water to prepare a primary suspension with a concentration of 10^−1^. Next, 1 mL of this initial suspension was transferred to 9 mL of sterile water and thoroughly mixed to obtain a 10^−2^ dilution. This serial dilution step was repeated to generate 10^−3^ and 10^−4^ soil suspensions. Aliquots of 200 μL from the 10^−3^ and 10^−4^ dilutions were separately spread uniformly on the surface of Rose Bengal Chloramphenicol (RBC) agar [34], which was supplemented with 0.03% streptomycin sulfate. The RBC agar medium had the following composition: peptone (5.00 g/L), glucose (10.00 g/L), MgSO_4_·7H_2_O (0.50 g/L), KH_2_PO_4_ (1.00 g/L), Rose Bengal (0.05 g/L), chloramphenicol (0.10 g/L), and agar (15.00 g/L). The inoculated RBC plates were incubated in darkness at 25 °C for 3–5 days. Subsequently, sporangia were manually picked using a sterile inoculating loop under a stereomicroscope (Olympus SZX10, Tokyo, Japan) and inoculated onto Potato Dextrose Agar (PDA) plates. The PDA medium was formulated as follows: 200 g potato, 20 g dextrose, 20 g agar, and 1000 mL distilled water, adjusted to pH 7.0. After inoculation, the PDA plates were incubated in darkness at 25 °C. Once purified, the isolated strains were preserved long-term in 10% glycerol at 4 °C.

Dried-type specimens were deposited in the Herbarium Mycologicum Academiae Sinicae (HMAS; Beijing, China). Ex-type strains were preserved in the China General Microbiological Culture Collection Center (CGMCC; Beijing, China) with duplicate cultures maintained at Shandong Normal University (XG; Jinan, China). Nomenclatural and taxonomic data pertaining to the new taxa have been submitted to both the Fungal Names Database (https://nmdc.cn/fungalnames/, accessed on 13 December 2015) and the National Microbiology Data Center (https://nmdc.cn/, accessed on 15 November 2015), in accordance with standard mycological archiving protocols.

### 2.2. Morphological Observation

Macroscopic morphological traits were documented using a high-definition color digital camera (DP80, Olympus; Tokyo, Japan), whereas microscopic characteristics were observed by means of a stereomicroscope (Olympus SZX10, Olympus; Tokyo, Japan) and an optical microscope (BX53, Olympus; Tokyo, Japan). Subsequently, Digimizer software (https://www.digimizer.com/, accessed on 20 October 2025) was applied to measure microstructures and Adobe Photoshop software (https://www.adobe.com/products/photoshop.html, accessed on 16 October 2025) was utilized for typesetting images.

### 2.3. DNA Extraction, PCR Amplification, and Sequencing

After the target strains were cultured on PDA plates at 25 °C for 5–7 days, the total genomic DNA was extracted from the mycelia using the BeaverBeads Plant DNA Kit [35] (Cat. No.: 70409–20; BEAVER Biomedical Engineering Co., Ltd.; Suzhou, China). The ITS, LSU rDNA, and *RPB1* regions were amplified by polymerase chain reaction (PCR) using the primer pairs ITS4/ITS5 [36], LR0R/LR5 [37], and f1843/R3096 [38], respectively (Table 1). PCR amplification was conducted in a total volume of 25 µL, consisting of 12.5 µL of 2× Hieff Canace^®^ Plus PCR Master Mix (Yeasen Biotechnology, Shanghai, China; Cat. No. 10154ES03), 10 µL of double-distilled water (ddH_2_O), 1 µL each of the 10 µM forward and reverse primers (TsingKe; Beijing, China), and 1 µL of fungal genomic DNA template. The amplification products were analyzed by 1% agarose gel electrophoresis, followed by visualization through staining with TS-GelRed Nucleic Acid Gel Stain (10,000× aqueous solution; Cat. No. TSJ002; TsingKe Biotech Co., Ltd.; Beijing, China). The target bands were recovered from the gel using the corresponding extraction kit (Cat. No. AE0101-C; Shandong Sparkjade Biotechnology Co., Ltd.; Jinan, China), and subsequent DNA sequencing was performed by Sangon Biotech (Shanghai, China). Consensus sequences were de novo assembled with Geneious Prime 2025.0.2 (https://www.geneious.com, accessed on 8 September 2025). All sequences were uploaded to the GenBank database for BLAST similarity searches and deposited under the accession numbers provided in Table S1.

### 2.4. Phylogenetic Analyses

The sequences used for phylogenetic analysis were obtained from the NCBI (https://www.ncbi.nlm.nih.gov/, accessed on 14 October 2025), and the details of these sequences are provided in Table S1. The multiple sequence alignment of each individual locus was performed via the mBed algorithm integrated with Clustal Omega 1.2.2 [39], followed by sequence concatenation using Geneious Prime 2025.0.2. Phylogenetic analyses were carried out based on the combined ITS-LSU-RPB1 sequence dataset, employing two complementary approaches: Maximum Likelihood (ML) and Bayesian Inference (BI). MrModeltest v.2.3 [40] was utilized to determine the optimal evolutionary model for each respective locus, and this model was subsequently incorporated into the BI analysis. For the ML analyses, RAxML 8.2.4 (https://www.phylo.org/, accessed on 14 October 2025) was employed with 1000 bootstrap replicates to assess tree reliability [41]. BI analyses were performed under parameters of rapid bootstrap configuration, 12 parallel chains, 5,000,000 generations, a sampling frequency of every 100 generations, and the auto-termination rule [42]. After discarding the first 25% of the initial samples (burn-in), the Posterior Probabilities were calculated based on the remaining trees. Finally, the phylogenetic tree was uploaded to the iTOL online platform (https://itol.embl.de, accessed on 20 October 2025) for optimization, and visually refined with Adobe Illustrator CC 2019 (https://adobe.com/products/illustrator, accessed on 20 October 2025).

## 3. Results

### 3.1. Phylogeny

**Figure 1:**
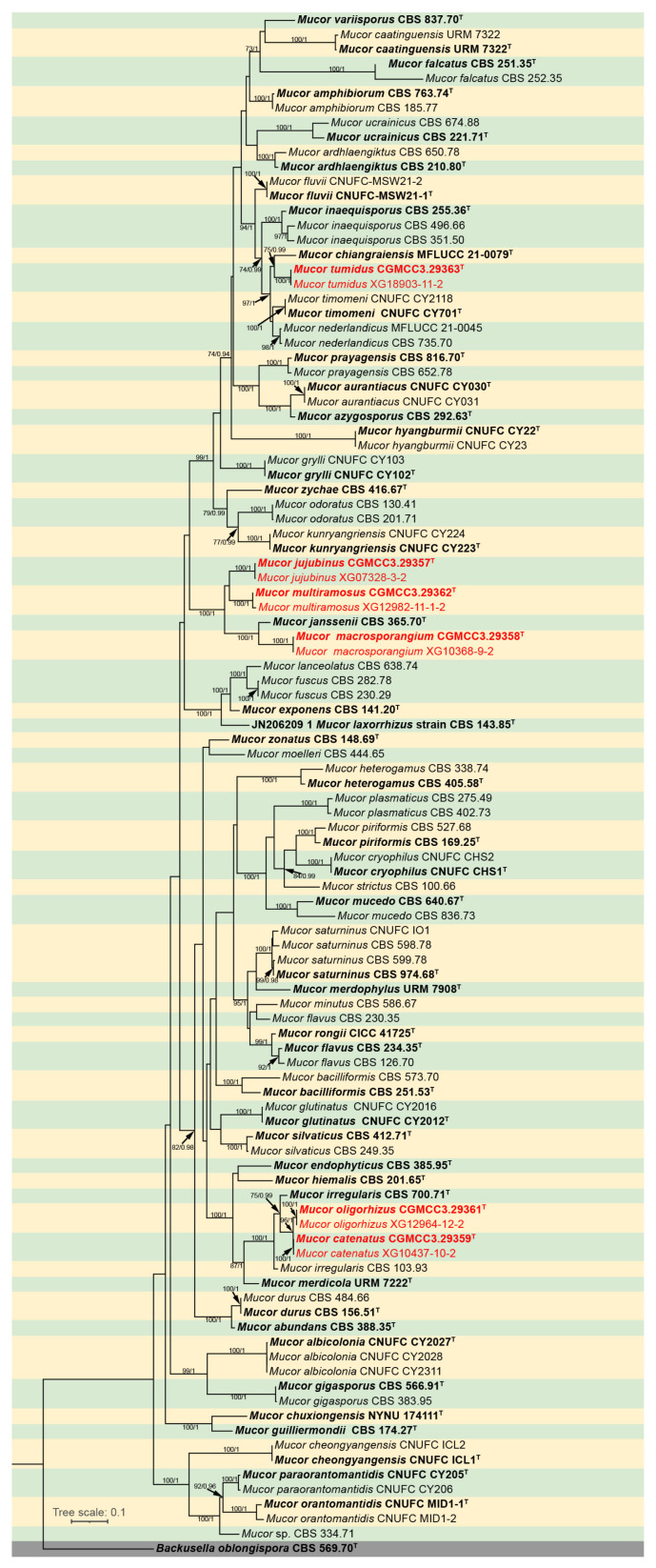
Maximum Likelihood (ML) phylogenetic tree of *Mucor* based on ITS, LSU, and *RPB1* sequences, with *Backusella oblongispora* as outgroup. Tree nodes are annotated with Maximum Likelihood Bootstrap Values (MLBV ≥ 70%) and Bayesian Inference Posterior Probabilities (BIPP ≥ 0.9), with the two values separated by a slash “/”. Ex-holotype strains are presented in bold font and marked with an asterisk “^T^”. Strains obtained in the present study are highlighted in red. The scale bar located in the lower-left corner corresponds to 0.1 nucleotide substitutions per site.

The molecular dataset included 105 strains in total, representing 60 *Mucor* species and an outgroup species *Backusella oblongispora*, consisted of 2837 characters, covering ITS rDNA (1–1116), LSU rDNA (1117–1812), and *RPB1* (1813–2837). Among these characters, 1564 were constant, 212 variables but parsimony uninformative, and 1061 parsimony informative. The results from MrModeltest demonstrated that the GTR+I+G evolutionary model, coupled with Dirichlet base frequencies, was suitable for all three partitions employed in the Bayesian Inference (BI) analyses. Given the topological congruence between the Maximum Likelihood (ML) tree and the BI tree, the ML tree was chosen as the representative topology for comprehensive visualization (Figure 1). Notably, the twelve *Mucor* strains isolated in this study formed six distinct, fully supported clades in the phylogenetic tree (Figure 1).

### 3.2. Taxonomy

**Figure 2:**
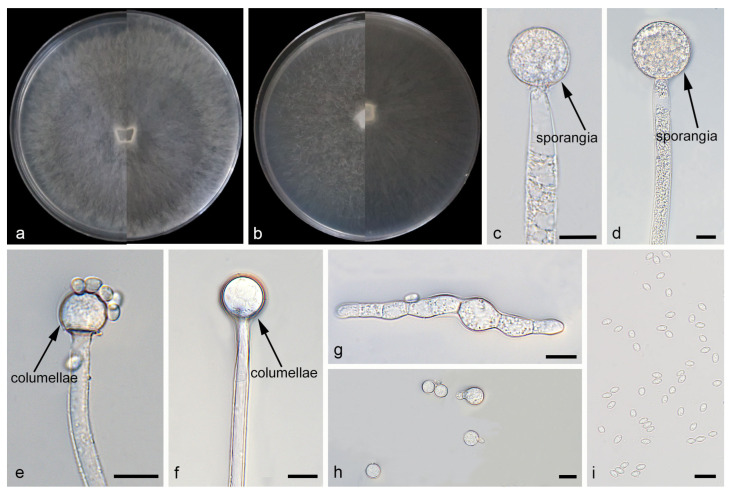
Morphologies of *Mucor catenatus* ex-holotype CGMCC 3.29359. (**a**) Obverse and reverse of the culture on PDA; (**b**) obverse and reverse of the culture on MEA; (**c**,**d**) sporangia; (**e**,**f**) columellae; (**g**,**h**) chlamydospores; and (**i**) sporangiospores. Scale bars: (**c**–**i**) 10 μm.

*Mucor catenatus*: W.X. Liu, H. Zhao, and X.Y. Liu, sp. nov.; Figure 2.

Fungal Names: FN 573147.

Type. China, Xizang Autonomous Region, Nyingchi City, Mainling City (29°04′26″ N, 94°23′85″ E, altitude 3165.71 m), from soil, 12 November 2024, W. X. Liu, holotype HMAS 354183, ex-holotype living culture CGMCC 3.29359 (=XG10437-10-1).

Etymology: The epithet *catenatus* (Lat.) refers to the species producing chlamydospores in chains.

Description. Colonies on MEA at 25 °C for 5 d, reaching 85 mm in diameter. Colonies on PDA at 25 °C for 5 d, reaching 85 mm in diameter, growing rapidly (17 mm/d). The colonies were white at the early stage, and their color shifted to yellowish-brown over time; floccose. Hyphae flourishing, radially growing, unbranched, hyaline, aseptate when juvenile, septate with age, 3.9–16.6 μm wide. Rhizoids absent. Stolons absent. Sporangiophores are produced by substrate as well as aerial hyphae, mostly erect, occasionally slightly bent, unbranched, hyaline, 3.0–18.3 μm wide. Sporangia globose, white to light brown, 16.0–65.3 μm in diameter. Collars may be present or absent; when present, they are typically conspicuous and well-developed with a relatively large size. Columellae globose or ovoid, hyaline or subhyaline, smooth-walled, 8.8–28.9 μm long and 8.2–27.8 μm wide. Apophyses absent. Sporangiospores fusiform, 2.4–6.5 μm long and 1.6–3.7 μm wide. Chlamydospores produced in substrate hyphae, in chains, globose, ellipsoid or irregular, 3.9–15.4 µm long and 3.3–10.4 µm wide. Zygospores unknown.

Additional strains examined. China, Xizang Autonomous Region, Nyingchi City, Mainling City (29°04′26″ N, 94°23′85″ E, altitude 3165.71 m), from soil, 12 November 2024, W. X. Liu, living culture XG10437-10-2; China, Xizang Autonomous Region, Nyingchi City, Mainling City (29°04′26″ N, 94°23′85″ E, altitude 3165.71 m), from soil, 26 October 2024, W. X. Liu, living culture XG10435-2; China, Xizang Autonomous Region, Nyingchi City, Mainling City (29°04′26″ N, 94°23′85″ E, altitude 3165.71 m), from soil, 26 October 2024, W. X. Liu, living culture XG10435-13; China, Fujian Province, Nanping City, Jianyang District, Shili Changjian (27°65′09″ N, 117°67′36″ E, altitude 445.3 m), from soil, 8 January 2025, W. X. Liu, living culture XG12147-9; China, Fujian Province, Nanping City, Wuyishan City, Xingcun Flower Sea Tourist Scenic Area (27°64′47″ N, 117°88′38″ E, altitude 239.5 m), from soil, 8 January 2025, W. X. Liu, living culture XG12179-11; China, Fujian Province, Sanming City, Yong’an City (25°89′13″ N, 117°49′21″ E, altitude 584.3 m), from soil, 24 December 2024, W. X. Liu, living culture XG12208-10; China, Fujian Province, Sanming City, Yong’an City (25°89′13″ N, 117°49′21″ E, altitude 584.3 m), from soil, 24 December 2024, W. X. Liu, living culture XG12208-11-1; China, Fujian Province, Sanming City, Yong’an City (25°89′13″ N, 117°49′21″ E, altitude 584.3 m), from soil, 24 December 2024, W. X. Liu, living culture XG12211-9; China, Fujian Province, Sanming City, Yong’an City (25°89′13″ N, 117°49′21″ E, altitude 584.3 m), from soil, 24 December 2024, W. X. Liu, living culture XG12211-10; China, Guangdong Province, Guangzhou City, Conghua District (23°64′07″ N, 113°76′57″ E, altitude 413.4 m), from soil, 31 July 2025, W. X. Liu, living culture XG15874-10; China, Guangdong Province, Guangzhou City, Conghua District, Y565 Township Road (23°62′42″ N, 113°80′04″ E, altitude 774.7 m), from soil, 8 July 2025, W. X. Liu, living culture XG15923-10; China, Guangdong Province, Guangzhou City, Conghua District, Y565 Township Road (23°62′42″ N, 113°80′04″ E, altitude 774.7 m), from soil, 8 July 2025, W. X. Liu, living culture XG15923-12; China, Guangdong Province, Guangzhou City, Conghua District, Y565 Township Road (23°62′42″ N, 113°80′04″ E, altitude 774.7 m), from soil, 8 July 2025, W. X. Liu, living culture XG15923-13; China, Guangdong Province, Qingyuan City, Yingde City (24°39′88″ N, 113°33′35″ E, altitude 160.2 m), from soil, 8 July 2025, W. X. Liu, living culture XG15933-12; China, Guangdong Province, Qingyuan City, Yingde City (24°39′88″ N, 113°33′35″ E, altitude 160.2 m), from soil, 18 July 2025, W. X. Liu, living culture XG15935-9; China, Yunnan Province, Xishuangbanna Dai Autonomous Prefecture, Jinghong City, Mengyang Town, G213 (22°14′62″ N, 100°88′96″ E, altitude 833.5 m), from soil, 20 August 2025, W. X. Liu, living culture XG18692-10; China, Yunnan Province, Xishuangbanna Dai Autonomous Prefecture, Jinghong City, Jinuo Ethnic Township, Jimeng Road (20°06′55″ N, 100°96′17″ E, altitude 0 m), from soil, 8 July 2025, W. X. Liu, living culture XG18732-12; China, Yunnan Province, Xishuangbanna Dai Autonomous Prefecture, Mengla County, Mengman Town, X171 (21°30′32,″ N, 101°30′04″ E, altitude 580.2 m), from soil, 8 July 2025, W. X. Liu, living culture XG18819-11; China, Zhejiang Province, Hangzhou City, Tonglu County, Yaolin Town (29°94′02,″ N, 119°53′24″ E, altitude 429.9 m), from soil, 20 August 2025, W. X. Liu, living culture XG20447-9; China, Zhejiang Province, Hangzhou City, Tonglu County, Yaolin Town (29°94′02,″ N, 119°53′24″ E, altitude 429.9 m), from soil, 20 August 2025, W. X. Liu, living culture XG20452-11; China, Zhejiang Province, Hangzhou City, Gongshu District, Banshan Street (30°35′82,″ N, 120°18′04″ E, altitude 135.7 m), from soil, 20 August 2025, W. X. Liu, living culture XG20252-12; China, Zhejiang Province, Hangzhou City, Tonglu County, Yaolin Town (29°94′02,″ N, 119°53′24″ E, altitude 429.9 m), from soil, 20 August 2025, W. X. Liu, living culture XG20457-9; China, Zhejiang Province, Hangzhou City, Tonglu County, Yaolin Town (29°94′02,″ N, 119°53′24″ E, altitude 429.9 m), from soil, 20 August 2025, W. X. Liu, living culture XG20457-12; China, Zhejiang Province, Hangzhou City, Tonglu County, Chengnan Street, Daqishan Road (29°76′56,″ N, 119°72′34″ E, altitude 153.4 m), from soil, 20 August 2025, W. X. Liu, living culture XG20482-9; China, Guizhou Province, Tongren City, Jiangkou County, County Road 585, near Shuiyuan Temple (27°82′90,″ N, 108°76′26″ E, altitude 481.6 m), from soil, 14 October 2025, W. X. Liu, living culture XG21026-12; China, Guizhou Province, Tongren City, Jiangkou County, County Road 585, near Shuiyuan Temple (27°82′90,″ N, 108°76′26″ E, altitude 481.6 m), from soil, 14 October 2025, W. X. Liu, living culture XG21033-9.

Notes. Based on the ITS–LSU–*RPB1* phylogenetic tree, strains CGMCC 3.29359 and XG10437-10-2 form a robust clade (MLBS/BIPP = 100/1.00; Figure 1), sister to *M. oligorhizus*. Morphologically, the two strains produce chlamydospores in chains, while chlamydospores are absent in *M. oligorhizus*. Based on morphological and molecular phylogenetic evidence, these two strains are proposed as a novel species *M. catenatus*.

**Figure 3:**
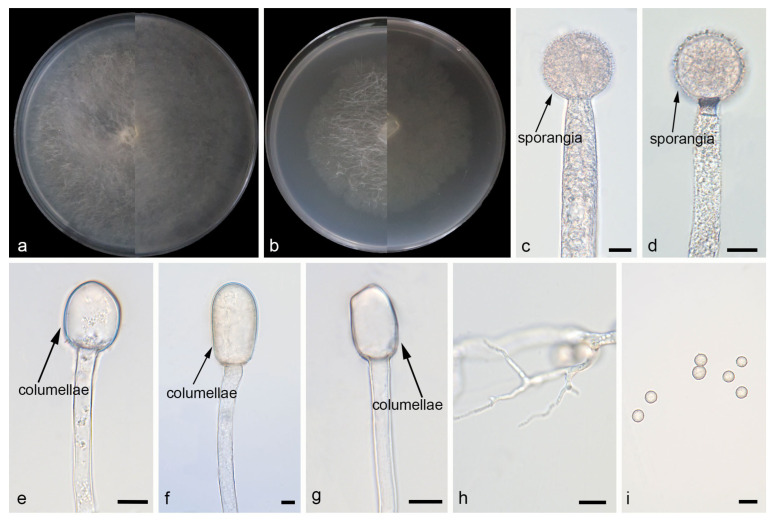
Morphologies of *Mucor jujubinus* ex-holotype CGMCC 3.29357. (**a**) Obverse and reverse of the culture on PDA; (**b**) obverse and reverse of the culture on MEA; (**c**,**d**) sporangia; (**e**–**g**) columellae; (**h**) rhizoids; and (**i**) sporangiospores. Scale bars: (**c**–**i**) 10 μm.

*Mucor jujubinus*: W.X. Liu, H. Zhao, and X.Y. Liu, sp. nov.; Figure 3.

Fungal Names: FN 573148.

Type. China, Hainan Province, Xinglong Tropical Botanical Garden (18°73′30″N, 110°19′87″E, altitude 25.6 m), from leaf, 25 April 2024, W. X. Liu, holotype HMAS 354181, ex-holotype living culture CGMCC 3.29357 (=XG07328-3-1).

Etymology: The epithet *jujubinus* (Lat.) refers to the species producing jujube-shaped columellae.

Description. Colonies on MEA at 25 °C for 5 d, reaching 64 mm in diameter. Colonies on PDA at 25 °C for 5 d, reaching 85 mm in diameter, growing rapidly (17 mm/d). The colonies were white at the early stage, and their color shifted to light brown over time; floccose. Hyphae flourishing, radially growing, unbranched, hyaline, aseptate when juvenile, septate with age, 2.1–15.1 μm wide. Rhizoids present but rare. Stolons absent. Sporangiophores are produced by substrate as well as aerial hyphae, usually erect, occasionally slightly bent, unbranched, hyaline, sometimes septate, 4.2–16.0 μm wide. Sporangia globose, pale yellow to light brown, smooth-walled, 21.5–44.7 μm in diameter. Collars absent. Apophyses absent. Columellae jujube-shaped, ovoid or ellipsoid, hyaline or subhyaline, 15.3–64.0 μm long and 10.6–46.6 μm wide. Sporangiospores globose, 3.5–9.4 μm in diameter. Chlamydospores absent. Zygospores unknown.

Additional strains examined. China, Hainan Province, Xinglong Tropical Botanical Garden (18°73′30″ N, 110°19′87″ E, altitude 25.6 m), from leaf, 25 April 2024, W. X. Liu, living culture XG07328-3-2.

Notes. Based on the ITS–LSU–*RPB1* phylogenetic tree, strains CGMCC 3.29357 and XG07328-3-2 form a robust clade (MLBS/BIPP = 100/1.00; Figure 1), sister to *M. multiramosus.* Morphologically, these two strains produce jujube-shaped columellae, while *M. multiramosus* is predominantly ovoid and ellipsoidal. Based on morphological and molecular phylogenetic evidence, these two strains are proposed as a novel species *M. jujubinus*.

**Figure 4:**
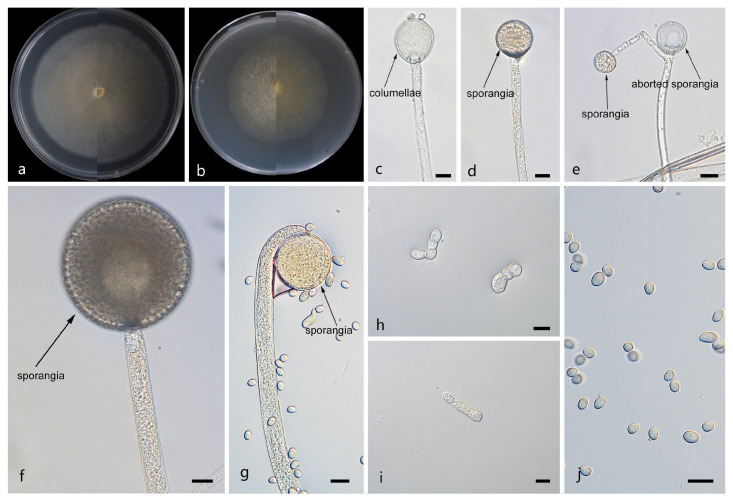
Morphologies of *Mucor macrosporangium* ex-holotype CGMCC 3.29358. (**a**) Obverse and reverse of the culture on PDA; (**b**) obverse and reverse of the culture on PDA; (**d**,**f**,**g**) sporangia; (**c**) columellae; (**e**) aborted sporangia; (**h**,**i**) chlamydospores; and (**j**) sporangiospores. Scale bars: (**c**–**j**) 10 μm.

*Mucor macrosporangium*: W.X. Liu, H. Zhao, and X.Y. Liu, sp. nov.; Figure 4.

Fungal Names: FN 573149.

Type. China, Xizang Autonomous Region, Nyingchi City, Medog County, near Xirang Village (29°18′52″ N, 95°03′35″ E, altitude 761.39 m), from soil, 15 November 2024, W. X. Liu, holotype HMAS 354182, ex-holotype living culture CGMCC 3.29358 (=XG10368-9-1)

Etymology: The epithet *macrosporangium* (Lat.) refers to the species producing macrosporangium.

Description. Colonies on MEA at 25 °C for 5 d, reaching 60 mm in diameter. Colonies on PDA at 25 °C for 5 d, reaching 70 mm in diameter, growing rapidly (14 mm/d). The colonies were white at the early stage, and their color shifted to yellowish-brown over time; floccose. Hyphae flourishing, radially growing, branched, hyaline, aseptate when juvenile, septate with age, 4.2–16.3 μm wide. Rhizoids absent. Stolons absent. Sporangiophores are produced by substrate as well as aerial hyphae, usually erect, occasionally slightly bent, unbranched, hyaline, 2.8–12.3 μm wide. Sporangia globose, pale yellow to dark brown, 14.5–63.4 μm in diameter. Aborted sporangia borne laterally on sporangiophores, 12.6–13.2 μm in diameter. Collars present or absent; if present, usually small. Columellae globose, ovoid or ellipsoid, hyaline or subhyaline, smooth-walled, 4.3–31.7 μm long and 2.2–27.5 μm wide. Apophyses absent. Sporangiospores fusiform or ellipsoid, 3.3–11.5 μm long and 2.4–6.4 μm wide. Chlamydospores are generated within the substrate hyphae, in chains, globose, ovoid, ellipsoid, or irregular, 7.5–11.1 µm long and 5.9–9.2 µm wide. Zygospores absent.

Additional strains examined. China, Xizang Autonomous Region, Nyingchi City, Medog County, near Xirang Village (29°18′52″ N, 95°03′35″ E, altitude 761.39 m), from soil, 15 November 2024, W. X. Liu, living culture XG10368-9-2; China, Xizang Autonomous Region, Nyingchi City, Medog County, near Xirang Village (29°18′52″ N, 95°03′35″ E, altitude 761.39 m), from soil, 15 November 2024, W. X. Liu, living culture XG10368-11.

Notes. Based on the ITS–LSU–*RPB1* phylogenetic tree, strains CGMCC 3.29358 and XG10368-9-2 form a robust clade (MLBS/BIPP = 100/1.00; Figure 1), sister to *Mucor janssenii* [43]. Morphologically, these two strains differ from *M*. *janssenii* by columellae and sporangiospores. The columellae of the new species are predominantly globose, ovoid, or ellipsoid, while those of *M. janssenii* predominantly obovoid, sometimes keyhole shaped. The sporangiospores in these two strains are larger than those in any others. Based on morphological and molecular phylogenetic evidence, these two strains are proposed as a novel species *M. macrosporangium*.

**Figure 5:**
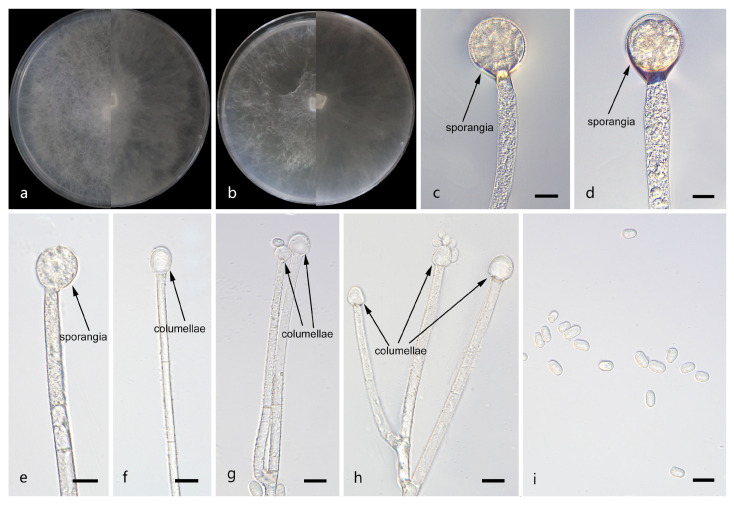
Morphologies of *Mucor multiramosus* ex-holotype CGMCC 3.29362. (**a**) Obverse and reverse of the culture on PDA; (**b**) obverse and reverse of the culture on MEA; (**c**,**d**) sporangia; (**e**–**h**) columellae; and (**i**) sporangiospores. Scale bars: (**c**–**i**) 10 μm.

*Mucor multiramosus*: W.X. Liu, H. Zhao, and X.Y. Liu, sp. nov.; Figure 5.

Fungal Names: FN 573150.

Type. China, Guangxi Zhuang Autonomous Region, Fangchenggang City, Shangsi County, Shiwandashan National Forest Park, northeastern corner (21°90′58″ N, 107°90′36″ E, altitude 277.0 m), from soil, September 2025, W. X. Liu, holotype HMAS 354186, ex-holotype living culture CGMCC 3.29362 (=XG12982-11-1-1).

Etymology: The epithet *multiramosus* (Lat.) refers to sporangiophores being highly branched.

Description. Colonies on MEA at 25 °C for 5 d, reaching 85 mm in diameter. Colonies on PDA at 25 °C for 5 d, reaching 85 mm in diameter, growing rapidly (17 mm/d). The colonies were white at the early stage, and their color shifted to yellow over time; floccose. Hyphae flourishing, radially growing, branched, hyaline, aseptate when juvenile, septate with age, 3.1–17.9 μm wide. Rhizoids absent. Stolons absent. Sporangiophores are produced by substrate as well as aerial hyphae, usually erect, occasionally slightly bent, solitary or with 2–3 branches, hyaline, occasionally septate, 2.8–10.9 μm wide. Fertile sporangia globose, pale yellow to yellowish-brown, 15.6–25.2 μm in diameter. Aborted sporangia borne terminally on sporangiophores, 13.3–13.5 μm in diameter. Collars absent. Columellae globose, ovoid or ellipsoid, hyaline or subhyaline, smooth-walled, 5.5–16.7 μm long and 7.2–17.5 μm wide. Apophyses absent. Sporangiospores usually ellipsoid, 3.4–9.8 μm long and 2.6–7.0 μm wide. Chlamydospores absent. Zygospores unknown.

Additional strains examined. China, Guangxi Zhuang Autonomous Region, Fangchenggang City, Shangsi County, Shiwandashan National Forest Park, northeastern corner (21°90′58″ N, 107°90′36″ E, altitude 277.0 m), from soil, 9 February 2025, W. X. Liu, living culture XG12982-11-1-2; China, Guangxi Zhuang Autonomous Region, Fangchenggang City, Shangsi County, Shiwandashan National Forest Park, northeastern corner (21°90′58″ N, 107°90′36″ E, altitude 277.0 m), from soil, 9 February 2025, W. X. Liu, living culture XG12982-12.

Notes. Based on the ITS–LSU–*RPB1* phylogenetic tree, strains CGMCC 3.29362 and XG12982-11-1-2 form a robust clade (MLBS/BIPP = 100/1.00; Figure 1), sister to *M. jujubinus.* Morphologically, sporangiophores are highly branched in these two strains, while *M. jujubinus* unbranched. Based on morphological and molecular phylogenetic evidence, these two strains are proposed as a novel species *M. multiramosus*.

**Figure 6:**
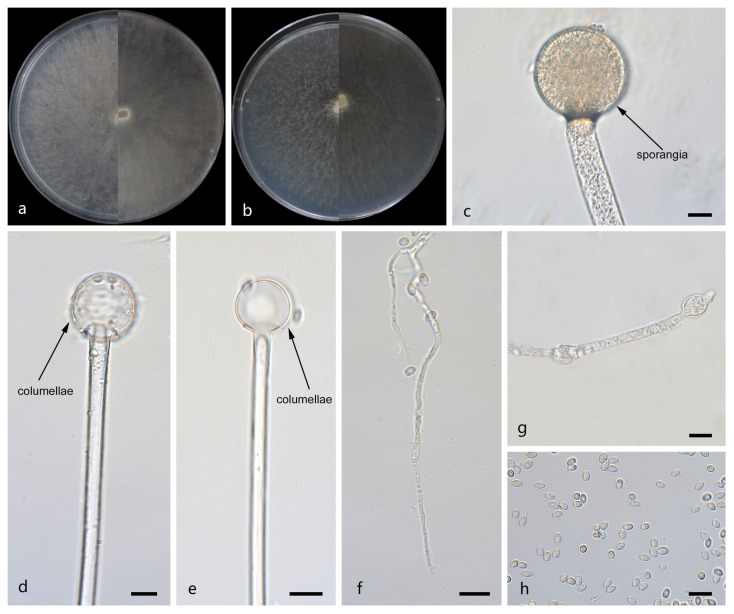
Morphologies of *Mucor oligorhizus* ex-holotype CGMCC 3.29361. (**a**) Obverse and reverse of the culture on PDA; (**b**) obverse and reverse of the culture on MEA; (**c**) sporangia; (**d**,**e**) columellae; (**f**) rhizoids; (**g**) swellings in hyphae; and (**h**) sporangiospores. Scale bars: (**c**–**h**) 10 μm.

*Mucor oligorhizus*: W.X. Liu, H. Zhao, and X.Y. Liu, sp. nov.; Figure 6.

Fungal Names: FN 573151.

Type. China, Guangxi Zhuang Autonomous Region, Nanning City, Xixiangtang District, Zhongliang Scenic Area (22°77′41″ N, 108°15′65″ E, altitude 84.7 m), from soil, 9 February 2025, W. X. Liu, holotype HMAS 354185, ex-holotype living culture CGMCC 3.29361 (=XG12964-12-1)

Etymology: The epithet *oligorhizus* (Lat.) refers to the species that have rare rhizoids.

Description. Colonies on MEA at 25 °C for 5 d, reaching 85 mm in diameter. Colonies on PDA at 25 °C for 5 d, reaching 85 mm in diameter, growing rapidly (17 mm/d). The colonies were white at the early stage, and their color shifted to light brown over time, floccose. Hyphae flourishing, radially growing, unbranched, hyaline, aseptate when juvenile, septate with age, 2.7–19.7 μm wide. Rhizoids usually absent, occasionally present. Stolons absent. Sporangiophores are produced by substrate as well as aerial hyphae, usually erect, occasionally slightly bent, unbranched, hyaline, sometimes accompanied by a swelling, 1.5–23.5 μm wide. Sporangia globose, brown, 15.0–81.7 μm in diameter. Collars absent. Columellae globose, ovoid or ellipsoid, hyaline or subhyaline, smooth-walled, 3.6–44.6 μm long and 3.6–43.9 μm wide. Apophyses absent. Sporangiospores usually fusiform, 2.2–5.3 μm long and 1.7–3.9 μm wide. Chlamydospores absent. Zygospores unknown.

Additional strains examined. China, Guangxi Zhuang Autonomous Region, Nanning City, Xixiangtang District, Zhongliang Scenic Area (22°77′41″ N, 108°15′65″ E, altitude 84.7 m), from soil, 9 February 2025, W. X. Liu, living culture XG12964-12-2; China, Guangxi Zhuang Autonomous Region, Nanning City, Xixiangtang District, Zhongliang Scenic Area (22°77′41″ N, 108°15′65″ E, altitude 84.7 m), from soil, 9 February 2025, W. X. Liu, living culture XG12963-10; China, Guangxi Zhuang Autonomous Region, Fangchenggang City, Fangcheng District, National Highway 219 (21°64′01″ N, 107°49′52″ E, altitude 664.3 m), from soil, 28 February 2025, W. X. Liu, living culture XG13003-11; China, Guangxi Zhuang Autonomous Region, Fangchenggang City, Fangcheng District, National Highway 219 (21°64′01″ N, 107°49′52″ E, altitude 664.3 m), from soil, 28 February 2025, W. X. Liu, living culture XG13006-11; China, Hainan Province, Wuzhishan City, Hongxia Valley Scenic Area (18°74′63″ N, 109°61′56″ E, altitude 367.2 m), from soil, 19 May 2025, W. X. Liu, living culture XG14839-11; China, Hainan Province, Qiongzhong Li and Miao Autonomous County, Baihualing Tropical Rainforest Tourism and Cultural Area (19°00′58″ N, 109°82′51″ E, altitude 392.4 m), from soil, 12 April 2025, W. X. Liu, living culture XG14866-13; China, Hainan Province, Qiongzhong Li and Miao Autonomous County, Baihualing Tropical Rainforest Tourism and Cultural Area (19°00′58″ N, 109°82′51″ E, altitude 392.4 m), from soil, 12 April 2025, W. X. Liu, living culture XG14869-10; China, Hainan Province, Qiongzhong Li and Miao Autonomous County, Baihualing Tropical Rainforest Tourism and Cultural Area (19°00′58″ N, 109°82′51″ E, altitude 392.4 m), from soil, 12 April 2025, W. X. Liu, living culture XG14869-12; China, Guangdong Province, Shaoguan City, Renhua County, Jinjiang Road (27°08′71″ N, 115°74′37″ E, altitude 108.2 m), from soil, 15 July 2025, W. X. Liu, living culture XG15947-9; China, Guangdong Province, Shaoguan City, Renhua County, Jinjiang Road (27°08′71″ N, 115°74′37″ E, altitude 108.2 m), from soil, 15 July 2025, W. X. Liu, living culture XG15947-11; China, Guangdong Province, Shaoguan City, Renhua County, Jinjiang Road (27°08′71″ N, 115°74′37″ E, altitude 108.2 m), from soil, 15 July 2025, W. X. Liu, living culture XG15947-13-1; China, Yunnan Province, Xishuangbanna Dai Autonomous Prefecture, Jinghong City, Mengyang Town, G213 (22°16′30″ N, 100°87′73″ E, altitude 765.9 m), from soil, 1 August 2025, W. X. Liu, living culture XG18697-12; China, Yunnan Province, Xishuangbanna Dai Autonomous Prefecture, Jinghong City, Mengyang Town (22°22′85″ N, 100°72′27″ E, altitude 572.1 m), from soil, 15 July 2025, W. X. Liu, living culture XG18720-11; China, Yunnan Province, Xishuangbanna Dai Autonomous Prefecture, Jinghong City, Mengyang Town (22°21′94″ N, 100°73′41″ E, altitude 578.2 m), from soil, 15 July 2025, W. X. Liu, living culture XG18731-11; China, Yunnan Province, Xishuangbanna Dai Autonomous Prefecture, Mengla County, Menglun Town, G213 (21°83′05″ N, 101°37′18″ E, altitude 772.2 m), from soil, 7 July 2025, W. X. Liu, living culture XG18760-9; China, Yunnan Province, Xishuangbanna Dai Autonomous Prefecture, Mengla County, Mengpeng Town (21°41′22″ N, 101°34′74″ E, altitude 544.2 m), from soil, 15 July 2025, W. X. Liu, living culture XG18806-13; China, Yunnan Province, Xishuangbanna Dai Autonomous Prefecture, Mengla County, Mengman Town, Mengman Service Area (21°31′80″ N, 101°29′81″ E, altitude 578.0 m), from soil, 4 July 2025, W. X. Liu, living culture XG18823-10; China, Yunnan Province, Xishuangbanna Dai Autonomous Prefecture, Mengla County, Mohan Town, G8511 (21°40′41″ N, 101°63′07″ E, altitude 639.8 m), from soil, 30 May 2025, W. X. Liu, living culture XG18847-10; China, Yunnan Province, Xishuangbanna Dai Autonomous Prefecture, Mengla County, Mohan Town, G8511 (21°40′41″ N, 101°63′07″ E, altitude 639.8 m), from soil, 30 May 2025, W. X. Liu, living culture XG18856-9; China, Yunnan Province, Xishuangbanna Dai Autonomous Prefecture, Mengla County, Mohan Town, G8511 (21°40′41″ N, 101°63′07″ E, altitude 639.8 m), from soil, 30 May 2025, W. X. Liu, living culture XG18856-11.

Notes. Based on the ITS–LSU–*RPB1* phylogenetic tree, strains CGMCC 3.29361 and XG12964-12-2 form a robust clade (MLBS/BIPP = 100/1.00; Figure 1), sister to *M. catenatus*. Morphologically, rhizoods are occasionally present in these two strains, but none in *M. catenatus*. Based on morphological and molecular phylogenetic evidence, these two strains are proposed as a novel species *M. oligorhizus*.

**Figure 7:**
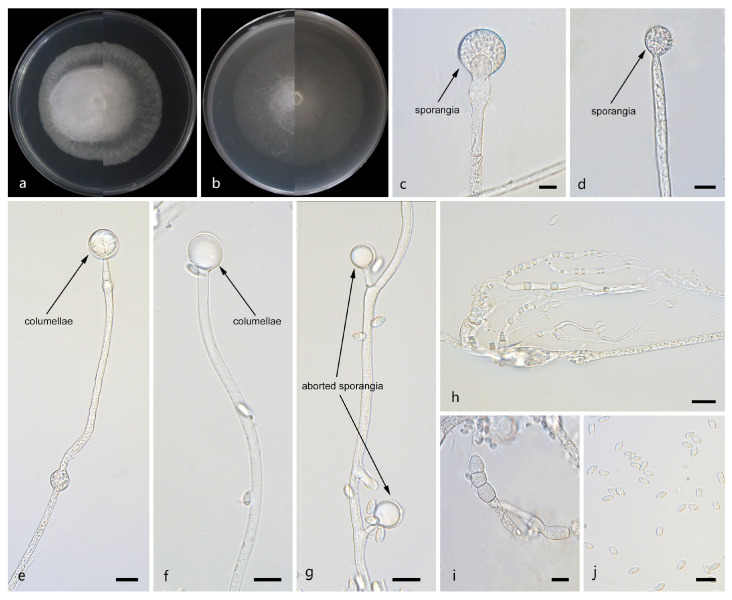
Morphologies of *Mucor tumidus* ex-holotype CGMCC 3.29363. (**a**) Obverse and reverse of the culture on PDA; (**b**) obverse and reverse of the culture on MEA; (**c**,**d**) sporangia; (**e**,**f**) columellae; (**g**) aborted sporangia; (**h**) rhizoids; (**i**) chlamydospores; and (**j**) sporangiospores. Scale bars: (**c**–**j**) 10 μm.

*Mucor tumidus*: W.X. Liu, H. Zhao, and X.Y. Liu, sp. nov.; Figure 7.

Fungal Names: FN 573152.

Type. China, Yunnan Province, Xishuangbanna Dai Autonomous Prefecture, Menghai County, Mengzhe Town, Menggang Highway (22°91′55″ N, 101°28′50″ E, altitude 1204.4 m), from soil, 19 May 2025, W. X. Liu, holotype HMAS 354187, ex-holotype living culture CGMCC 3.29363 (=XG18903-11-1).

Etymology: The epithet *tumidus* (Lat.) refers to the swollen sporangiophores.

Description. Colonies on MEA at 25 °C for 5 d, reaching 62 mm in diameter. Colonies on PDA at 25 °C for 5 d, reaching 58 mm in diameter, growing rapidly (11.6 mm/d). The colonies were white at the early stage, and their color shifted to pale yellow over time, floccose. Hyphae flourishing, radially growing, unbranched, hyaline, aseptate when juvenile, septate with age, 2.1–15.3 μm wide. Rhizoids present. Stolons absent. Sporangiophores are produced by substrate as well as aerial hyphae, usually erect, occasionally slightly bent, unbranched, hyaline, sometimes accompanied by a swelling, occasionally septate, 2.5–18.3 μm wide. Fertile sporangia globose, white to light gray, 8.6–27.0 μm in diameter. Aborted sporangia borne laterally on aerial hyphae, 7.7–23.4 μm in diameter. Collars absent. Columellae globose or ovoid, hyaline or subhyaline, smooth-walled, 5.9–20.5 μm long and 5.1–17.9 μm wide. Apophyses absent. Sporangiospores usually fusiform, 3.5–7.3 μm long and 1.9–3.2 μm wide. Chlamydospores are generated within the aerial hyphae, in chains, irregular, 6.5–29.3 μm long and 6.5–22.7 μm wide. Zygospores unknown.

Additional strains examined. China, Yunnan Province, Xishuangbanna Dai Autonomous Prefecture, Menghai County, Mengzhe Town, Menggang Highway (22°91′55″ N, 101°28′50″ E, altitude 1204.4 m), from soil, 19 May 2025, W. X. Liu, living culture XG18903-11-2; China, Yunnan Province, Xishuangbanna Dai Autonomous Prefecture, Menghai County, Mengzhe Town, Menggang Highway (22°91′55″ N, 101°28′50″ E, altitude 1204.4 m), from soil, 19 May 2025, W. X. Liu, living culture XG18903-10.

Notes. Based on the ITS–-LSU-*RPB1* phylogenetic tree, strains CGMCC 3.29363 and XG18903-11-2 form a robust clade (MLBS/BIPP = 100/1.00; Figure 1), sister to *Mucor chiangraiensis* [44]. Morphologically, these two strains are distinguished from *M. chiangraiensis* in sporangiophores and sporangia. These two strains have swellings in sporangiophores and aborted sporangia, while *M. chiangraiensis* does not. Based on morphological and molecular phylogenetic evidence, these two strains are proposed as a novel species *M. tumidus*.

## 4. Discussion

Based on molecular phylogenetic studies of ITS, LSU, and *RPB1*, as well as traditional morphological classification, this study reports six new species in *Mucor* (*M. catenatus* sp. nov., *M. jujubinus* sp. nov., *M. macrosporangium* sp. nov., *M. multiramosus* sp. nov., *M. oligorhizus* sp. nov., and *M. tumidus* sp. nov.) from soil collected in southern China. They are all form their own clades with full supports on multi-locus phylogram (*M. catenatus* MLBS/BIPP = 100/1.00, *M. jujubinus* MLBS/BIPP = 100/1.00, *M. macrosporangium* MLBS/BIPP = 100/1.00, *M. multiramosus* MLBS/BIPP = 100/1.00, *M. oligorhizus* MLBS/BIPP = 100/1.00, and *M. tumidus* MLBS/BIPP = 100/1.00; Figure 1). A comparative analysis of the morphological characteristics of these six new species and their closely related species was conducted and is shown in Table 2.

*Mucor jujubinus* and *M. multiramosus* are sister groups to each other; meanwhile, *M. jujubinus* and *M. multiramosus* are closely related to *M. janssenii*. *M. jujubinus* possesses large, date-shaped columellae and unbranched sporangiophores, while *M. multiramosus* exhibits multibranched sporangiophores and occasionally aborted sporangia. Compared with *M. janssenii*, *M. jujubinus* produces larger sporangiospores; additionally, the columellae of *M*. *janssenii* are keyhole shaped, whereas those of *M. jujubinus* are date shaped. In comparison, *M. multiramosus* differs from *M. janssenii* in that the former has branched sporangiophores while the latter has unbranched ones. *M. macrosporangium* and *M. janssenii* are also sister groups. *M. macrosporangium* produces larger sporangia and more abundant chlamydospores, while *M. janssenii* does not produce any chlamydospores. *M. catenatus* is sister to *M. oligorhizus*; meanwhile, *M. catenatus*, *M. oligorhizus*, and *M. merdicola* share a common node in the phylogenetic tree, indicating a close phylogenetic relationship. *M. catenatus* produces chlamydospores in chains and lacks rhizoids, while chlamydospores are absent in *M. oligorhizus*, with well-developed rhizoids; by comparison, the chlamydospores of *M. merdicola* are globose, subglobose, or doliform, and its rhizoids are poorly developed. Meanwhile, the sporangiophores of *M. catenatus* and *M. oligorhizus* are unbranched, whereas those of *M. merdicola* are branched. *M. tumidus* is closely related to *M. chiangraiensis.* In *M. tumidus*, the sporangiophore occasionally swells and bears lateral, aborted sporangia, whereas in *M. chiangraiensis* it does not.

Mucorales fungi are distributed worldwide and are commonly found in soil. The *Mucor* is the most species-rich genus within the class Mucorales. Over the past three years, at least 30 new species of *Mucor* have been discovered (http://www.indexfungorum.org/, accessed on 28 September 2025) [1,29,38]. The six new species identified in this study increase the total number of globally recognized *Mucor* species to 60, contributing to the understanding of *Mucor* diversity in the world, especially in China.

However, with the rapid development of molecular biology technologies, multi-gene phylogenetic tree construction has become a core method for new species identification, phylogenetic resolution, and taxonomic delimitation. Compared with single-gene analysis, it effectively avoids biases from differential evolutionary rates of individual genes and better reflects interspecific evolutionary histories. However, some *Mucor* strains in this study have missing or incomplete sequences of key molecular markers (RPB1, ITS, and LSU), which exclude them from multi-gene phylogenetic analyses. This reduces the topological stability of phylogenetic trees, limits the precise resolution of interspecific relationships, and thus causes obvious limitations in molecular systematic research regarding species coverage and the reliability of results. Meanwhile, morphological research also has shortcomings: the core taxonomic characteristics of some *Mucor* strains are described only briefly, lacking quantitative data and clear morphological atlas records. This impairs accurate morphological comparisons between different strains, and between target strains and known species, which weakens the supporting role of morphological traits in species identification and differentiation. Ultimately, it exacerbates difficulties in *Mucor* species identification and classification, and hinders the accurate delimitation of new species.

## Figures and Tables

**Table 1:** PCR primers and programs used in this study.

Loci	PCR Primers	Primer Sequence (5′–3′)	PCR Cycles	References
ITS	ITS5	GGA AGT AAA AGT CGT AAC AAG G	95 °C 5 min; (95 °C 30 s, 55 °C 30 s, 72 °C 1 min) × 35 cycles; and 72 °C 10 min	[36]
	ITS4	TCC TCC GCT TAT TGA TAT GC		
LSU	LR0R	GTA CCC GCT GAA CTT AAG C	95 °C 5 min; (95 °C 50 s, 47 °C 30 s, 72 °C 1.5 min) × 35 cycles; and 72 °C 10 min	[37]
	LR5	TCC TGA GGG AAA CTT CG		
*RPB1*	f1843	ATT TYG AYG GTG AYG ARA TGA AC	94 °C 30 s; (51 °C 30 s, 72 °C 1 min) × 5 cycles; (49 °C 30 s, 72 °C 1 min) × 5 cycles; (47 °C 30 s, 72 °C 1 min) × 5 cycles; and 72 °C 10 min	[38]
	R3096	GRA CRG TDC CRT CAT AYT TRA CC		

**Table 2:** Comparisons of morphological characteristics of *Mucor* species proposed in this study and their allies.

Species	Colonies	Sporangiophores	Sporangia	Columellae	Sporangiospores	Chlamydospores	References
*M. catenatus*	PDA: 25 °C 5 d, 85 mm, 17 mm/d, initially white, and gradually becoming yellowish-brown floccose.	Unbranched; 3.0–18.3 μm wide.	Globose, white to light brown, and 16.0–65.3 μm diameter.	Globose, ovoid; 8.8–28.9 × 8.2–27.8 μm.	Fusiform; 2.4–6.5 × 1.6–3.7 μm.	In chains, globose, ellipsoid, or irregular, and 3.9–15.4 × 3.3–10.4 µm.	This study.
*M. chiangraiensis*	PDA: 25 °C 3 d, 48 mm, 16 mm/d, white, and floccose.	Unbranched or sympodial branches; 12 μm in diameter.	globose, hyaline to pale brown, and 18–40.5 × 18.5–40.5 µm.	Subglobose, obovoid, or ellipsoid; 12.5–23.5 × 11.5–23 µm.	Ellipsoidal, oval, and 4–6.5 × 2–3.5 µm.	Irregular.	[44]
*M. janssenii*	NA	NA	NA	Obovoid; keyhole shaped.	6 μm	NA	[43]
*M. jujubinus*	PDA: 25 °C 5 d, 85 mm, 17 mm/d, initially white, gradually becoming light brown, and floccose.	Unbranched; 4.2–16.0 μm wide.	Globose, pale yellow to light brown, and 21.5–44.7 μm diameter.	Ovoid, ellipsoid, and 15.3–64.0 × 10.6–46.6 μm.	Globose; 3.5–9.4 μm diameter.	Absent.	This study.
*M. macrosporangium*	PDA: 25 °C 5 d, 70 mm, 14 mm/d, initially white, gradually becoming yellowish-brown, and floccose.	Unbranched; 2.8–12.3 μm wide.	Globose, pale yellow to dark brown, and 14.5–63.4 μm diameter.	Globose, ovoid, ellipsoid, and 4.3–31.7 × 2.2–27.5 μm.	Fusiform, ellipsoid, 3.3–11.5 and × 2.4–6.4 μm.	In chains, globose, ovoid, or ellipsoid; 7.5–11.1 × 5.9–9.2 µm.	This study.
*M. merdicola*	Initially MEA: 25 °C 4 d, 95 mm, 23.8 mm/d white, gradually becoming yellowish to cream, and floccose.	Simple or repeatedly sympodially branched; 3–18 μm wide.	Globose, yellow to grayish brown, and 16–85 μm diameter.	Globose, subglobose, and applanate; 15–29 × 30–35.	Ellipsoid, fusiform, and 2.5–7 × 5–10.5.	Globose, subglobose, and doliform.	[45]
*M. multiramosus*	PDA: 25 °C 5 d, 85 mm, 17 mm/d, initially white, and gradually becoming yellow floccose.	Branched; 2.8–10.9 μm wide.	Globose, pale yellow to yellowish-brown, and 15.6–25.2 μm diameter.	Globose, ovoid, or ellipsoid; 5.5–16.7 × 7.2–17.5 μm.	Ellipsoid; 3.4–9.8 × 2.6–7.0 μm.	Absent.	This study.
*M. oligorhizus*	PDA: 25 °C 5 d, 85 mm, 17 mm/d, initially white, and gradually becoming light brown floccose.	Unbranched; 1.5–23.5 μm wide.	Globose, brown, and 15.0–81.7 μm diameter.	Globose, ovoid, ellipsoid; 3.6–44.6 × 3.6–43.9 μm.	Fusiform; 2.2–5.3 × 1.7–3.9 μm.	Absent.	This study.
*M. tumidus*	PDA: 25 °C 5 d, 58 mm, 11.6 mm/d, initially white, and gradually becoming yellow floccose.	Unbranched; 2.5–18.3 μm wide.	Globose, white to light gray, and 8.6–27.0 μm diameter.	Globose or ovoid; 5.9–20.5 × 5.1–17.9 μm.	Fusiform; 3.5–7.3 × 1.9–3.2 μm.	In chains, irregular, and 6.5–29.3 × 6.5–22.7 μm.	This study.

Notes: “NA”, data are not available in the representative references.

## Data Availability

All sequences were uploaded to the GenBank database; the accession numbers are provided in Table S1. The original contributions presented in this study are included in the article and Supplementary Material. Further inquiries can be directed to the corresponding author.

## Supplementary Materials

The following supporting information can be downloaded at https://www.mdpi.com/article/10.3390/jof12020098/s1. Table S1: GenBank accession numbers of *Mucor* and *Backusella* strains in this study.
