# Supplementary material for: Unveiling Species Diversity Within Early-Diverging Fungi from China XII: Six New Species of Mucor (Mucoromycota)
Source: J Fungi (Basel). 2026 Jan 30;12(2):98. doi: 10.3390/jof12020098 (PMC12942298; doi:10.3390/jof12020098)
Supplement: Supplementary file 1 [file jof-12-00098-s001.zip › jof-4033178-supplementary.pdf]

Table S1. GenBank accession numbers of *Mucor* and *Backusella* strains in this study.

Taxon Name	Strain Number	GenBank accession numbers		
		ITS	LSU	*rpb1*
Mucor abundans	CBS 388.35 T	JN206111	NA	MT500100
Mucor amphibiorum	CBS 185.77	JN206170	NA	NA
Mucor amphibiorum	CBS 763.74 T	HM999957	HM849688	MT500115
Mucor albicolonia	CNUFC CY2027 T	PP844894	PP851426	NA
Mucor albicolonia	CNUFC CY2028	PP844895	PP851427	NA
Mucor albicolonia	CNUFC CY2311	PP844896	PP851428	NA
Mucor aurantiacus	CNUFC CY030 T	PP844897	PP851429	PP893222
Mucor aurantiacus	CNUFC CY031	PP844898	PP851430	PP893223
Mucor azygosporus	CBS 292.63 T	JN206187	JN206497	MT500118
Mucor ardhlaengiktus	CBS 210.80 T	JN206172	JN206504	MT500117
Mucor ardhlaengiktus	CBS 650.78	JN206174	JN206499	NA
Mucor bacilliformis	CBS 251.53 T	JN206083	JN206451	MT500067
Mucor bacilliformis	CBS 573.70	JN206084	JN206452	NA
Mucor caatingaensis	URM 7322 T	KT960377	KT960369	MT500113
Mucor caatingaensis	URM 7322	KT960376	KT960370	NA
Mucor catenatus	**CGMCC 3.29359 T**	**PX443276**	**PX443337**	**NMDCN0009FO8**
Mucor catenatus	**XG10437-10-2**	**PX443277**	**PX443338**	**NMDCN0009R83**
Mucor cheongyangensis	CNUFC ICL1 T	MN592639	MN592643	NA
Mucor cheongyangensis	CNUFC ICL2	MN592640	MN592644	NA
Mucor chiangraiensis	MFLUCC 21-0079 T	MZ433253	MZ433250	NA
Mucor cryophilus	CNUFC CHS1 T	PP844923	PP852708	PP886119
Mucor cryophilus	CNUFC CHS2	NA	PP852709	PP886120
Mucor chuxiongensis	NYNU 174111 T	NR_185548	NG_228784	NA
Mucor durus	CBS 156.51 T	JN206112	JN206456	MT500101
Mucor durus	CBS 484.66	JN206113	NA	NA
Mucor endophyticus	CBS 385.95 T	JN206159	JN206448	MT500068
Mucor exponens	CBS 141.20 T	JN206206	JN206441	MT500051
Mucor falcatus	CBS 251.35 T	JN206250	JN206509	NA
Mucor falcatus	CBS 252.35	JN206249	NA	NA
Mucor flavus	CBS 230.35	JN206061	JN206464	MT500088
Mucor flavus	CBS 234.35 T	JN206051	JN206468	MT500091
Mucor flavus	CBS 126.70	JN206049	JN206469	MT500092
Mucor fluvii	CNUFC-MSW21-2	MF667991	MF667996	NA
Mucor fluvii	CNUFC-MSW21-1 T	MF667992	MF667995	NA
Mucor fuscus	CBS 282.78	JN206201	JN206442	NA
Mucor fuscus	CBS 230.29	JN206204	NA	MT500053
Mucor gigasporus	CBS 566.91 T	JN206247	JN206494	NA
Mucor gigasporus	CBS 383.95	JN206246	NA	NA
Mucor glutinatus	CNUFC CY2012 T	PP844899	PP852710	PP886121
Mucor glutinatus	CNUFC CY2016	PP844900	PP852711	PP886122
Mucor grylli	CNUFC CY102 T	OM868230	OM843127	NA

Mucor grylli	CNUFC CY103	NA	OM843128	NA
Mucor guiliermondii	CBS 174.27 T	JN206082	JN206475	MT500064
Mucor heterogamus	CBS 405.58 T	JN206167	JN206487	NA
Mucor heterogamus	CBS 338.74	JN206169	JN206488	NA
Mucor hiemalis	CBS 201.65 T	JN206125	HM849683	MT500073
Mucor hyangburmii	CNUFC CY22 T	OM868232	OM843129	NA
Mucor hyangburmii	CNUFC CY23	NA	OM843129	NA
Mucor inaequisporus	CBS 255.36 T	JN206177	JN206502	MT500119
Mucor inaequisporus	CBS 496.66	JN206179	JN206501	NA
Mucor inaequisporus	CBS 351.50	JN206178	JN206500	NA
Mucor irregularis	CBS 700.71 T	JN206154	JN206450	MT500065
Mucor irregularis	CBS 103.93	JN206150	HM849684	NA
Mucor janssenii	CBS 365.70	MH859713	MH871465	MF495212
Mucor jujubinus	**CGMCC 3.29357 T**	**PX443280**	**PX443341**	**NMDCN0009FOA**
Mucor jujubinus	**XG07328-3-2**	**PX443281**	**PX443342**	**NMDCN0009R85**
Mucor kunryangriensis	CNUFC CY223 T	OM868234	OM843131	NA
Mucor kunryangriensis	CNUFC CY224	NA	OM843132	NA
Mucor lanceolatus	CBS 638.74	JN206205	JN206443	MT500055
Mucor laxorrhizus	CBS 143.85 T	JN206209	JN206444	MT500052
Mucor macrosporangium	**CGMCC 3.29358 T**	**PX443278**	**PX443339**	**NMDCN0009FO9**
Mucor macrosporangium	**XG10368-9-2**	**PX443279**	**PX443340**	**NMDCN0009R85**
Mucor merdicola	URM 7222 T	KT960374	KT960372	MT500070
Mucor merdophylus	URM 7908 T	MK775467	MK775466	NA
Mucor minutus	CBS 586.67	JN206048	JN206463	MT500085
Mucor moelleri	CBS 444.65	JN206114	HM849682	MT500098
Mucor mucedo	CBS 836.73	JN206092	NA	NA
Mucor mucedo	CBS 640.67 T	JN206085	HM849687	MT500079
Mucor multiramosus	**CGMCC 3.29362 T**	**PX443270**	**PX443331**	**NMDCN0009FO6**
Mucor multiramosus	**XG12982-11-1-2**	**PX443271**	**PX443332**	**NMDCN0009R81**
Mucor nederlandicus	CBS 735.70	JN206176	JN206503	MT500120
Mucor nederlandicus	MFLUCC 21-0045	MZ433254	MZ433251	NA
Mucor odoratus	CBS 130.41	JN206197	JN206495	MT500042
Mucor odoratus	CBS 201.71	JN206198	NA	NA
Mucor oligorhizus	**CGMCC 3.29361 T**	**PX443272**	**PX443333**	**NMDCN0009FO7**
Mucor oligorhizus	**XG12964-12-2**	**PX443273**	**PX443334**	**NMDCN0009R82**
Mucor orantomantidis	CNUFC-MID1-1 T	MH594737	MH591457	NA
Mucor orantomantidis	CNUFC-MID1-2	MH594738	MH591458	NA
Mucor paraorantomantidis	CNUFC CY205 T	PP844901	PP851431	PP893224
Mucor paraorantomantidis	CNUFC CY206	PP844902	PP851432	PP893225
Mucor plasmaticus	CBS 275.49	JN206078	JN206483	MT500084
Mucor plasmaticus	CBS 402.73	JN206081	NA	NA
Mucor piriformis	CBS 169.25 T	JN206028	HM849681	MT500077
Mucor piriformis	CBS 527.68	JN206034	JN206476	NA
Mucor prayagensis	CBS 652.78	JN206189	JN206498	NA

Mucor prayagensis	CBS 816.70 T	JN206188	JN206496	MT500111
Mucor rongii	CICC 41725 T	MK903014	MK903013	MT815282
Mucor saturninus	CBS 598.78	JN206074	NA	NA
Mucor saturninus	CBS 974.68 T	JN206072	JN206458	MT500086
Mucor saturninus	CBS 599.78	JN206073	NA	NA
Mucor saturninus	CNUFC IO1	PP843591	PP851433	PP886123
Mucor silvaticus	CBS 249.35	JN206122	JN206455	NA
Mucor silvaticus	CBS 412.71 T	JN206124	NA	MT500066
Mucor sp.	CBS 334.71	JN206248	JN206518	MT500056
Mucor strictus	CBS 100.66	JN206035	JN206477	NA
Mucor timomeni	CNUFC CY701 T	PP844926	PP892773	PP893226
Mucor timomeni	CNUFC CY2118	NA	PP892774	PP893227
Mucor tumidus	**CGMCC 3.29363 T**	**PX443268**	**PX443329**	**NMDCN0009FO5**
Mucor tumidus	**XG18903-11-2**	**PX443269**	**PX443330**	**NMDCN0009R80**
Mucor ucrainicus	CBS 674.88	JN206192	JN206507	NA
Mucor ucrainicus	CBS 221.71 T	JN206191	NA	MT500044
Mucor variisporus	CBS 837.70 T	JN206175	JN206508	MT500114
Mucor zonatus	CBS 148.69 T	JN206104	JN206454	MT500105
Mucor zychae	CBS 416.67 T	JN206199	JN206505	NA
Mucor janssenii	CBS 365.70 T	MH859713.1	MH871465.1	MF495212.1
Backusella oblongispora	CBS 569.70 T	JN206251	JN206407	OP832481

Notes: New species discovered herein are shown in bold. The asterisk "T" indicates the ex-type or ex-holotype strains. The "NA" stands for "not available".
